# A case report: Veno-venous extracorporeal membrane oxygenation for severe blunt thoracic trauma

**DOI:** 10.1186/s13019-019-0908-9

**Published:** 2019-05-06

**Authors:** Fumihiro Ogawa, Takuma Sakai, Ko Takahashi, Makoto Kato, Keishi Yamaguchi, Sayo Okazaki, Takeru Abe, Masayuki Iwashita, Ichiro Takeuchi

**Affiliations:** 1 Department of Emergency Medicine, Yokohama City University School of Medicine, Yokohama, 232-0024 Japan; 2 Advanced Critical Care and Emergency Center, Yokohama City University Medical Center, Yokohama, 232-0024 Japan; 3 Department of Emergency Medicine, Yokohama City University Graduate School of Medicine, Yokohama, 232-0024 Japan

**Keywords:** Veno-venous extracorporeal membrane oxygenation, Blunt trauma, Hemopneumothorax

## Abstract

**Introduction:**

The use of veno-venous extracorporeal membrane oxygenation (VV-ECMO) in trauma patients has been controversial, but VV-ECMO plays a crucial role when the lungs are extensively damaged and when conventional management has failed. VV-ECMO provides adequate tissue oxygenation and an opportunity for lung recovery. However, VV-ECMO remains contraindicated in patients with a risk of bleeding because of systemic anticoagulation during the treatment. The most important point is controlling the bleeding from severe trauma.

**Case:**

A 32-year-old male experienced blunt trauma due to a traffic accident. He presented with bilateral hemopneumothorax and bilateral flail chest. We performed emergency thoracotomy for active bleeding and established circulatory stability. After surgery, the oxygenation deteriorated under mechanical ventilation, so we decided to establish VV-ECMO. However, bleeding from the bilateral lung contusions increased after VV-ECMO was established, and the patient was switched to heparin-free ECMO. After conversion, we could control the bronchial bleeding, especially the lung hematomas, and the oxygenation recovered. The patient was discharged without significant complications. VV-ECMO and mechanical ventilation were stopped on days 10 and 11, respectively. He was discharged from the ICU on day 15.

**Conclusion:**

When we consider the use of ECMO for patients with uncontrollable, severe bleeding caused by blunt trauma, it may be necessary to use a higher flow setting for heparin-free ECMO than typically used for patients without trauma to prevent thrombosis.

## Introduction

Blunt trauma caused by traffic accidents is occasionally a lethal problem. Patients with blunt trauma reportedly experience associated chest trauma in 50% of cases [1, 2]. Life-threatening complications include hemorrhagic shock and severe respiratory failure due to chest trauma [3]. Additionally, the role of extracorporeal life support (ECLS) in trauma patients remains unclear, although the first-ever successful application of ECLS was to treat posttraumatic acute respiratory distress syndrome in 1971 [4]. Several case reports and small case series have described the use of ECLS in trauma patients with various injury patterns and mixed outcomes [1, 4–9]. However, ECLS remains infrequently utilized in this patient population due in large part to concern regarding the risk of major hemorrhage [9]. Larger database studies have confirmed that ECLS is infrequently used in trauma patients; however, hospital survival is reported to be 44% to as high as 74.1% [10–15], similar to the reported 58% survival in the general adult respiratory ECLS population [16]. Extracorporeal membrane oxygenation (ECMO), a type of ECLS, helps maintain systemic tissue oxygenation when pulmonary function is compromised. However, ECMO is contraindicated in some patients, particularly in those where further bleeding may be induced by the systemic anticoagulation involved in treatment, for example, patients with hemorrhagic blunt trauma associated with pulmonary contusions and other organ damage [8]. Therefore, attention must be paid to the potential for increased bleeding and coagulopathy. The application of heparin-free ECMO may be a solution for systemic anticoagulation during treatment. There have been few cases of ECMO application in patients with massive hemothorax due to deep lung lacerations. Here, we report the successful use of heparin-free ECMO in a 32-year-old male who experienced respiratory failure due to extensive bilateral lung damage.

### Case

**Fig. 1:**
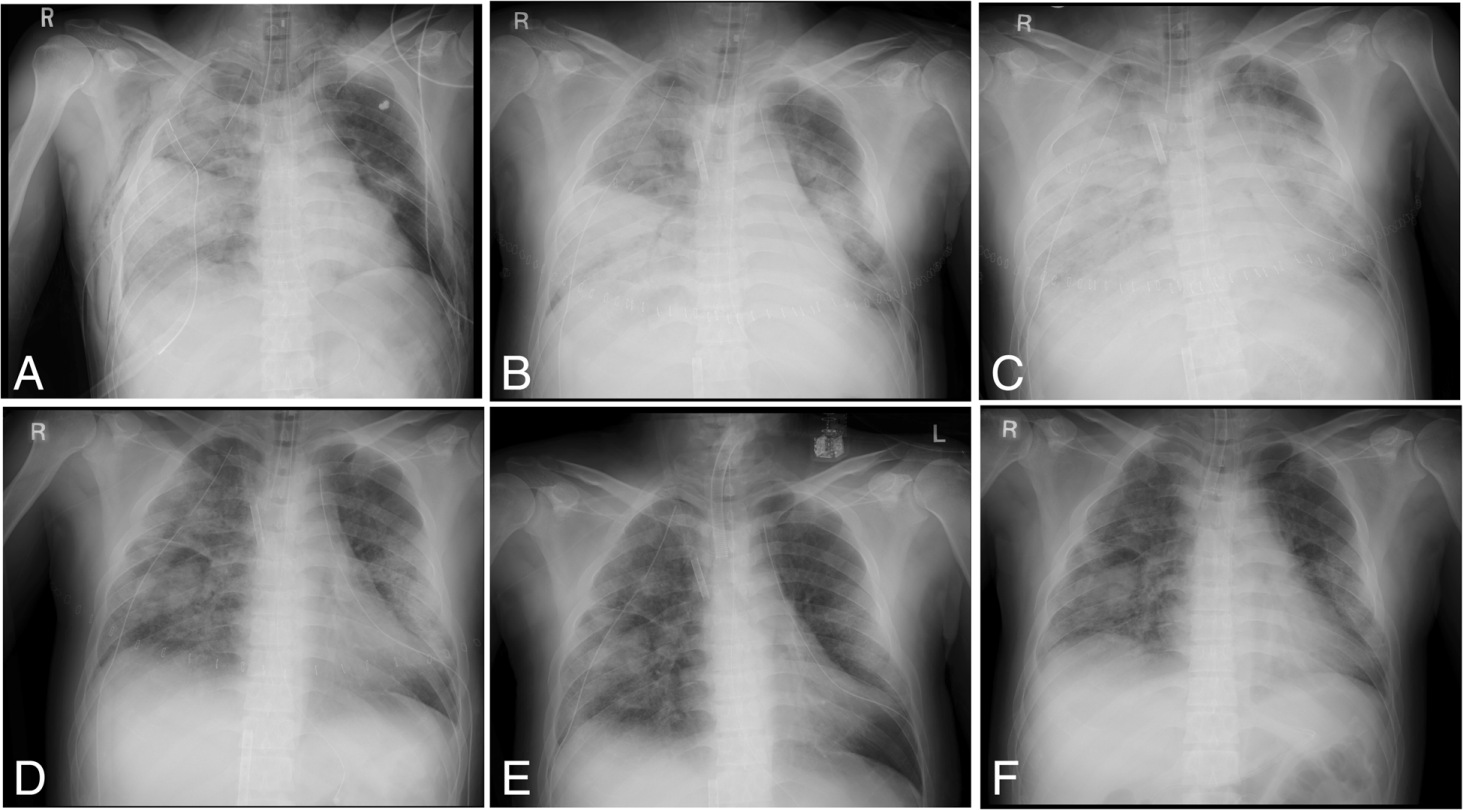
Chest X-ray images during the course of treatment: **a** initial; **b** postthoracotomy; **c** day 3 under full-heparin ECMO; **d** day 8 after heparin-free ECMO; **e** day 10 after removing ECMO; **f** day 15 after removing thoracic tubes

**Fig. 2:**
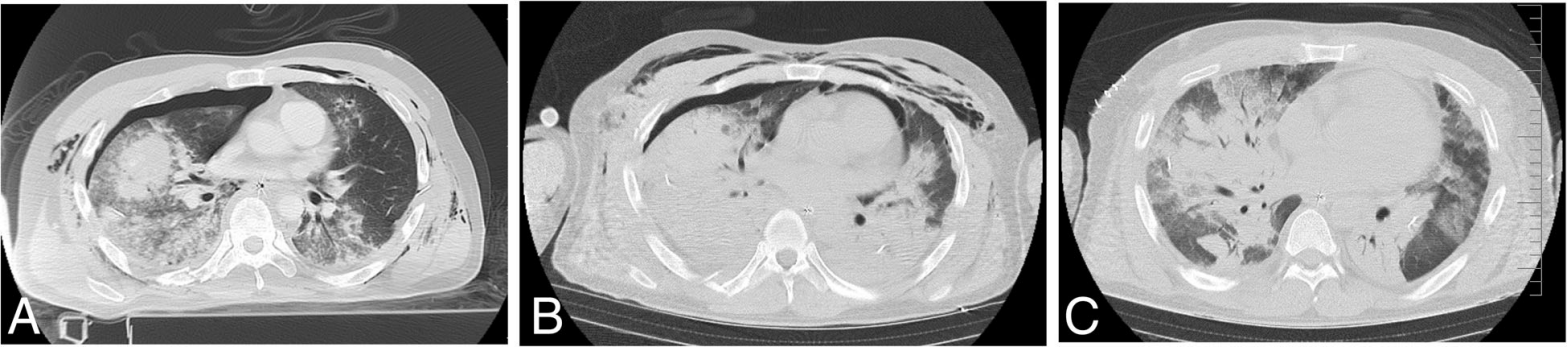
Chest CT images during the course of treatment: **a** initial; **b** postthoracotomy; **c** day 8 after heparin-free ECMO. Bleeding from bilateral lung contusions decreased before and after heparin-free ECMO

A 32-year-old male experienced blunt trauma due to a traffic accident riding a motorcycle stuck by a truck. Then, he was admitted to our emergency department by an ambulance. At the time of arrival at our emergency department, he was conscious without any motor deficits. Clinical examination revealed severe hypoxia with SpO_2_ 70% at 10 L/min O_2_, tachypnea at 42 breaths/min, and tachycardia at 154 beats/min with severe hypotension, 54/24. Breathing sounds were decreased, and flail chest and severe subcutaneous emphysema of the entire upper body were observed at the initial evaluation, as revealed by chest computed tomography (CT), brain CT, and a focused assessment with sonography for trauma (FAST) performed as early as possible. There were no intracranial hemorrhages or definitive abdominal organ injuries. Simple chest radiography and chest CT showed large bilateral hemothorax with atelectasis and severe contusions in both lungs (Figs. 1a, 2a). His blood pH, PaO_2_, and PaCO_2_ were 7.30, 84.3 mmHg (oxygen saturation, 96%), and 47.8 mmHg on a reservoir mask at 10 L/min oxygen, respectively. We diagnosed spinous process fractures at C6 and C7 (abbreviated injury scale (AIS); 2 pts), right lateral rib fractures at 1–11, left lateral rib fractures at 1–3, 5, and 7, bilateral lung contusions, bilateral hemothorax (AIS; 5 pts), a right clavicle fracture (AIS; 2 pts), and a left scapula fracture (AIS; 2 pts). The injury severity score was 33 and the probability of survival was 0.72.

**Fig. 3:**
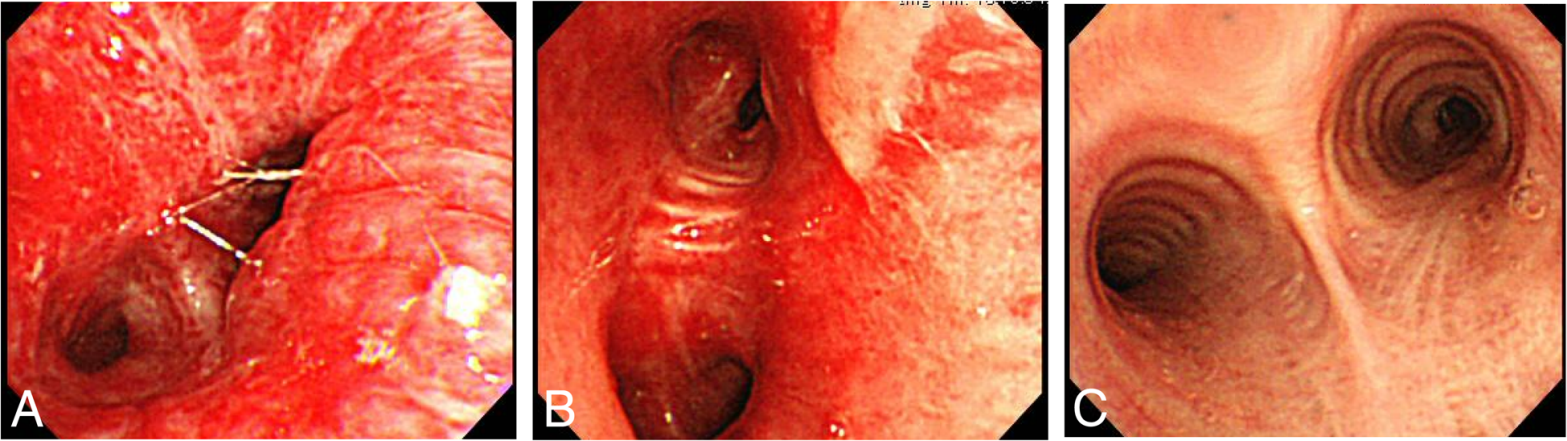
Bronchoscopy images during the course of treatment: **a** day 3 under full-heparin ECMO; **b** day 5 after heparin-free ECMO; **c** day 8 after heparin-free ECMO, with decreased bleeding from lung contusions

At admission, we performed intubation and thoracic drainage for hemothorax. Then, we took the patient to an operating room to achieve surgical hemostasis of the bilateral hemothorax by clamshell thoracotomy for massive bleeding from chest drainage tube. We identified pleural lacerations, so we initiated the control of active bleeding there. Severe respiratory failure due to lung contusions persisted at the time of the patient’s admission to the ICU (Figs. 1b, 2b). He gradually gained increased invasiveness by mechanical ventilation, so chest x-ray showed decreasing lung roentgen lucent; the PaO_2_/FiO_2_ ratio (PFR) was 112 mmHg with a positive end expiratory pressure (PEEP) of 20 cm H_2_O, and after thoracotomy, at an inspiration pressure of 33 cm H_2_O, arterial blood gas analysis showed that the pH, PaO_2_, and PaCO_2_ were 7.41, 73.1 mmHg (oxygen saturation, 96%), and 45.5 mmHg, respectively. Therefore, we decided to establish veno-venous (VV)-ECMO (CAPIOX SP-200 TERUMO Cardiovascular Systems, Tokyo, Japan) and performed cannulation via the right jugular vein (18-Fr cannula for inflow, Toyobo, Tokyo, Japan) and the right femoral vein (24-Fr cannula for outflow, Toyobo, Tokyo, Japan). We set the mechanical ventilation at a lower pressure. After we established VV-ECMO (1800 rpm; pump flow, 4 L/min; O_2_ flow, 2 L/min) with standard heparin for ECMO, the bronchial bleeding and bleeding from the bilateral lung contusions increased. Therefore, we needed to check and aspirate the bleeding by endotracheal bronchoscopy (Fig. 3a). However, the bleeding was very severe because full heparinization (activated coagulation time (ACT) range, 180–200 s) for ECMO was used. A chest x-ray and CT scan showed increased severity of the lung contusions and hemorrhages, indicating acute respiratory disorder syndrome (Figs. 1c, 2c). Therefore, we decided to continue ECMO without heparin due to the severe bleeding while being careful of blood coagulation without other substitute anticoagulation drugs.

**Fig. 4:**
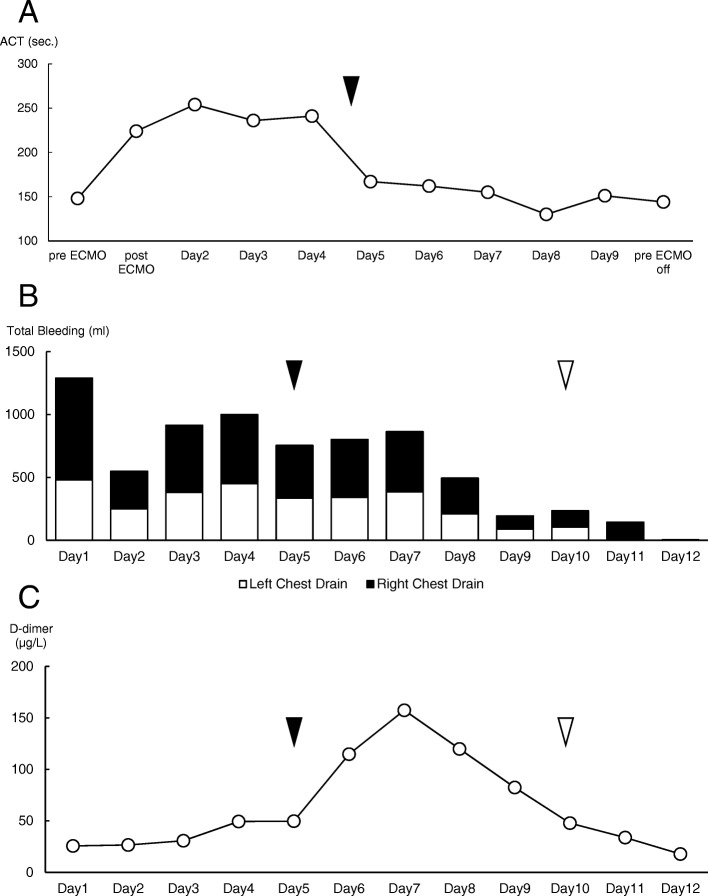
Graphs of metrics during the course of treatment: **a** ACT; **b** total bleeding from chest drainage tubes. Black bar: right chest drain. White bar: left chest drain. **c** D-dimer. We converted to heparin-free ECMO on day 5 (black arrowhead) and stopped ECMO on day 10 (white arrowhead)

After conversion, the ACT normalized (Fig. 4a), and the bleeding from the chest drains decreased gradually (Fig. 4b) instead of D-dimer increased gradually (Fig. 4c). Then, we could control the bleeding from the lung contusions and bronchus (Fig. 3b), and both the lung hematomas and oxygenation recovered (Fig. 2d). During this period of VV-ECMO, some treatments could be performed without any issues related circuit thrombosis and oxygenation failure. We performed tracheostomy on day 9 following ventilation with a PEEP of 20 cm H_2_O and an inspiratory pressure of 30 cm H_2_O because he needed ventilation support after removing VV-ECMO because of severe chest trauma and ARDS when we checked normalized coagulation after canceling anticoagulation before ECMO weaning. Subsequently, ECMO weaning was initiated because all of his underlying diseases were removed and improvement of lung function (FiO_2_ < 0.35, PEEP < 10 cmH_2_O, PFR ≥ 250) on his spontaneous breathing after the extracorporeal blood flow was stepwise reduced to 1.5 L/min., then Gas flow is tapered mostly in parallel to the blood flow and finally shut off for 30–60 min without dyspnea or tachypnea, and VV-ECMO was stopped on day 10 (Fig. 1e). There were no ECMO-related complications during the course of treatment. We removed the chest drains on day 11 (left) and day 12 (right). Mechanical ventilation weaning was initiated on day 12, and he was discharged from the ICU on day 15 (Fig. 1f).

## Discussion

In this time, we described the case we experienced a severe blunt trauma patients using with heparin-free ECMO for massive bleeding after traffic accident. We can find many heparin-free ECMO reports for severe blunt trauma. But all of them were retrospective observational study or cohort study, not case report. So, we described a detailed case report for severe chest blunt trauma with chest x-ray, bronchoscopy images and laboratory data. Severe trauma causes approximately 5 million deaths annually worldwide [3, 17]. Many patients respond well to specialized trauma care treatments, including fluid resuscitation, mechanical ventilation, and other invasive procedures. However, patients with concurrent severe chest trauma and hemorrhagic shock have a poor prognosis. The significant treatment goals for patients with severe blunt chest trauma and hemorrhagic shock are restoring blood coagulation via appropriate transfusions (red blood cells, platelets and fresh frozen plasma), surgically repairing areas of bleeding, and maintaining body temperature.

The potential survival benefit of ECMO applied in patients with severe lung injury has recently been reported [5, 6]. We believe that if there is no hemorrhaging in organs other than the lungs, the application of ECMO will likely have a low risk of causing additional hemorrhage. However, if there is hemorrhaging in other organs, the application of ECMO should be cautiously considered depending on whether any of the additional hemorrhaging can be controlled. Early ECMO initiation carries a theoretically increased risk of ECMO-related complications in trauma patients, most notably hemorrhage. Trauma-induced coagulopathy is a well-described process associated with significant morbidity and mortality rates [18–21]. Hemorrhage is a significant concern in trauma patients, and bleeding complications are seen in 35–59% of trauma patients treated with ECMO [7, 9]. Specialized patient management strategies, including initiating heparin-free ECMO and titrating the ACT goals based on bleeding risk, have been described in an effort to minimize bleeding risk in post-trauma ECMO patients [3, 7, 8]. Advancements in ECMO technology within the last decade, including heparin-coated circuitry and polymethylpentene oxygenators, have decreased thrombogenicity and therefore mitigated anticoagulation requirements in certain clinical scenarios [22]. These technological advancements allow for individualized bleeding assessments and subsequent alterations to anticoagulation parameters in trauma patients, with minimal anticoagulation as a possibility if necessary [3, 8]. In such cases, heparin-free ECMO should also be considered. Similarly, if there is active hemorrhaging from lung contusions or bronchus, regardless of hemorrhage in other organs, careful consideration of ECMO is needed to account for the control of possible hemorrhage. We understood heparin-free ECMO was acceptable for severe trauma patients in spite of worse survival [10]. So, we focused on blood flow of ECMO because of preventing thrombosis and clot formation. In our case, considering the possibility of thrombus formation with during heparin-free ECMO applied for lung rest, we set the blood flow rate higher than the usual blood flow used for ECMO to prevent thrombosis. During this period, it is very important to check coagulation factors, ACT, APTT, PT and D-dimer, especially D-dimer is more sensitive marker for thrombus formation. In this case, D-dimer value was gradually elevated after canceling anticoagulation as we expected. But D-dimer was not over cut-off value for thrombus formation, so he had no thrombus formation and no complication for VV-ECMO. Therefore, considering the use of ECMO to improve oxygenation in patients with severe trauma and hemorrhage that is difficult to control, we recommend that 1) heparin, which promotes bleeding in ECMO, be stopped and that 2) higher blood flow rate settings than usually used for non-trauma patients be considered to prevent thrombosis in the ECMO circuit. From the above, we considered about the new information for severe blunt trauma.

Damage control focused on bleeding, and stable vital signs were maintained. Matthias et al. have reported that the use of heparin-free ECMO is beneficial for the survival of blunt trauma patients with pulmonary failure and hemorrhagic shock [3]. Although contraindicated in blunt trauma patients with hemorrhagic shock, surgical repair followed the application of ECMO may be feasible if bleeding is well controlled. In this case, we used ECMO because the patient had no irreversible injuries and bleeding control was maintained after the thoracotomy. The outcome revealed no ventilator-induced barotrauma and no bleeding complications. On the other hand, prolonged heparin-free ECMO has been applied successfully in patients with severe head injury or traumatic brain injury (TBI) [11, 23]. It is certainly possible that with higher numbers, the presence of TBI will independently correlate with worse outcomes for patients on ECMO for traumatic lung failure. We believe that the use of heparin has a clinically significant impact, and larger samples sizes are needed to further characterize this relationship, especially in those with TBI. In this way, ECMO has some risk for severe trauma patients, so ECMO support may not be the first treatment option in patients with traumatic lung contusion with alveolar hemorrhage, and its use is even contested in injured, bleeding patients. However, in a patient with severe traumatic lung injury and alveolar hemorrhage with intractable hypoxemia and hypercapnia, ECMO merits consideration and may be key to survival in this situation.

## Conclusion

ECMO may serve as an additional treatment modality in adult patients with severe traumatic lung injury or acute respiratory failure that does not respond to maximal conventional ventilation support. However, heparin-free ECMO in a patient with severe blunt chest trauma and coexisting hemorrhagic shock suggests that ECMO can be a safe and highly effective rescue treatment under more careful observation.

## Abbreviations

ACT: activated coagulation time
AIS: abbreviated injury scale
CT: computed tomography
ECLS: extracorporeal life support
ECMO: extracorporeal membrane oxygenation
FAST: focused assessment with sonography with sonography for for trauma
ICU: intensive care unit
ISS: injury severity score
PEEP: positive end expiratory pressure
TBI: traumatic brain injury
VV-ECMO: veno-venous extracorporeal membrane oxygenation
